# A new approach of presenting reversible logic gate in nanoscale

**DOI:** 10.1186/s40064-015-0928-4

**Published:** 2015-03-31

**Authors:** Ali Newaz Bahar, Sajjad Waheed, Nazir Hossain

**Affiliations:** Department of Information and Communication Technology, Mawlana Bhashani Science and Technology University, Tangail, 1902 Bangladesh; Department of Physics, University of South Dakota, Vermillion, SD 57069 USA

**Keywords:** Quantum-dot Cellular Automata (QCA), Complementary Metal Oxide Semiconductor (CMOS), Nanoscale reversible gate

## Abstract

Conventional lithography-based VLSI design technology deployed to optimize low-powered-computing and higher scale integration of semiconductor components. However, this downscaling trend confronts serious challenges of tunneling and leakage current increment to the Complementary Metal–Oxide–Semiconductor (CMOS) technology on nanoscale regimes. To resolve the physical restriction of the CMOS, Quantum-dot Cellular Automata (QCA) technology dedicates for the nanoscale technology that embrace a new information transformation technique. However, QCA is limited to the design of the sequential and combinational circuits only. This paper presents some highly scalable features reversible logic gate for the QCA technology. In addition, proposed layout compared with CMOS technology, offer a better reduction in size up to 233 times.

## Introduction

Over the years, the reversible logic has attained a great attention due to their ability of power minimization which is the main requirement in the low power VLSI design. This technology is a promising computing paradigm that has immense applications in emerging technologies such as quantum dot cellular automata, quantum computing, optical computing, DNA computing, optical information processing, etc. (Al-Rabadi 2004; Ma et al. 2008; Thapliyal and Ranganathan 2008; Thapliyal and Ranganathan 2009a; Thapliyal and Ranganathan 2010). In reversible circuits the input and output mapping is one-to-one that means every unique output vector is generated from each input vector, and vice versa. It has shown by (Landauer 1961) that the loss of every bit of information dissipates energy of *k*T*ln*2 joules, where *k* is Boltzmann’s constant and *T* is the absolute temperature. In room temperature *T*_*R*_, the amount of heat generated due to one bit of information loss (Landauer 1961) is small, which is calculated as 2.9 × 10^−21^ joule, but is not negligible. Later on, (Bennett 1973) showed that the energy losses could be avertable; if the computation is carried out by reversible circuits.

Now-a-days, CMOS technology is imminent to its physical boundary in downscaling and confronting critical challenges of designing ultra low power consuming computational devices. This projected the expectation to go looking new technologies that offer emerging solutions. One of the alternatives is known as Quantum-dot Cellular Automata (QCA) (Lent et al. 1993a; Lent et al. 1993b) which has recently been recognized as one of the top emerging technologies with potential applications in future computing (Orlov et al. 1997; Wilson et al. 2002) for its express speed, nanoscale integration and ultra low power consumption in various computational applications (Lent et al. 1993a).

Molecular QCA can operate at room temperature shown in (Lent et al. 2003; Wang and Lieberman 2004). Since the emancipation of QCA, a number of QCA-based logic circuits have been proposed based on majority voter gate, inverter and QCA wires. A lot of QCA based combinational (Azghadi et al. 2007; Cho and Swartzlander 2007; Cho and Swartzlander 2009; Gin et al. 1999; Hänninen and Takala 2010; Ke-ming and Yin-shui 2007; Kim et al. 2007; Mardiris and Karafyllidis 2010; Navi et al. 2010; Sara et al. 2012; Sayedsalehi et al. 2011; Srivastava and Bhanja 2007; Tougaw and Lent 1994; Vetteth et al. 2002; Wang et al. 2003; Zhang et al. 2005), sequential (Askari et al. 2008; Dehkordi et al. 2011; Ghosh et al. 2013; Huang et al. 2007; Sen et al. 2013; Vankamamidi et al. 2008; Venkataramani et al. 2008; Wu et al. 2014; Xiao et al. 2012; Yang et al. 2010) circuits have been proposed in recent years. However, reversible logic circuit designs (Bahar et al. 2013; Shah et al. 2012) in QCA are still unexplored research area. In this paper, four novel QCA circuit layouts of reversible logic gate have been presented and their functionality has been verified using the QCADesigner (Walus et al. 2004).

## Material and methods

**Figure 1:**
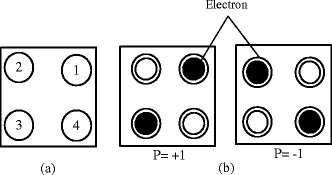
**Basic structure of a QCA cell with four dots (a), different positions of the electrons based on polarization (b).**

A Quantum Cellular Automata, one of the emerging nanotechnologies was first introduced by (Lent et al. 1993a) which encodes information based on position of electrons. The basic element of a QCA based device is the squared cell with two mobile electrons and two quantum dots (Amlani et al. 1999; Ling-gang et al. 2005) shown in Figure 1. Based on the occupied electron's position, a QCA cell has two different types of polarization, *P* = +1 or binary 1 and *P* = -1 or binary 0 (Lent and Tougaw 1997). A cell polarization p is +1 if the electrons are occupied the position 1 and 3, similarly a cell polarization p is -1 in the case of electrons are occupied the position 2 and 4. The equation for the cell polarization (Lent and Tougaw 1997) is given below:

1 $$ P=\frac{\left({\rho}_2+{\rho}_4\right)-\left({\rho}_1+{\rho}_3\right)}{\left({\rho}_1+{\rho}_2+{\rho}_3+{\rho}_4\right)} $$

Where, *ρ*_*i*_ denotes the electronic charge at dot *i*.

**Figure 2:**
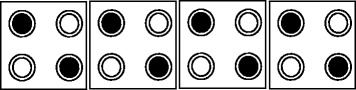
**QCA wire.**

The QCA based design consists of a wire, a 3-input majority voter gate, and an inverter. An array of cells arranged one after another makes up the QCA wire, as shown in Figure 2. In the QCA wire, the polarization of each cell is affected by the electrostatic forces generated through neighboring cells. Thus, information propagates from one cell to another by through the QCA wires.

**Figure 3:**
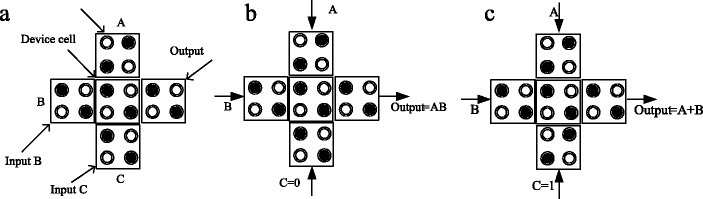
**The QCA majority gate (a), function as (b) the AND gate and (c) the OR gate.**

The 3-input majority gate has five cells: three inputs, a middle cell, and one output shown in the Figure 3 (a). The middle cell of the 3 input majority gates switches major polarization and maintains a consistent output. If the polarization of one of the 3-input cells is constant to *P* = -1 or *P* = +1 then this gate can be programmed to function as a 2-input AND or a 2-input OR gates, respectively shown in the Figure 3 (b) and (c).

**Figure 4:**
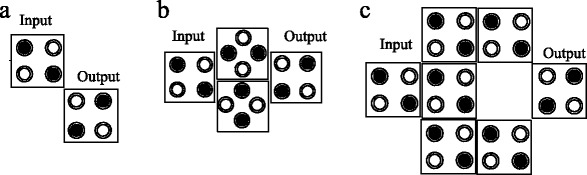
**Three different structure of inverter gates (a) two cell inverter (b) four cell inverter (c) seven cell inverter.**

In the Figure 4 shows the variety module of the inverting gate in the QCA. Seven cells inverter in the Figure 4 (c) operate appropriately in all various circuits.

## Proposed circuits and presentation

A reversible logic gate is one that has *n* input *n* output; with one-to-one mapping that means it determines the outputs from the inputs. It also helps the inputs to be uniquely recovered or reconstructed from the outputs.

### NFT gate

**Figure 5:**
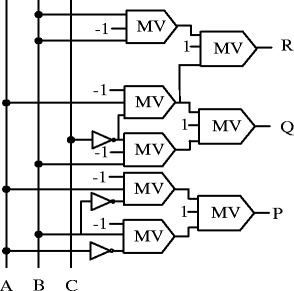
**Proposed QCA block diagram of NFT gate.**

The New Fault Tolerant (NFT) gate is one of the basic 3 × 3 parity preserving (Haghparast and Navi 2008) reversible logic gates having the inputs and output mapping as P = A ⊕ B, $\mathrm{Q}=\overline{\mathrm{B}}\mathrm{C}\oplus \mathrm{A}\overline{\mathrm{C}}$ and $\mathrm{R}=\mathrm{B}\mathrm{C}\oplus \mathrm{A}\overline{\mathrm{C}}$, where the input vector is ***I*** (*A*, *B*, *C*) and the output vector is ***O*** (*P*, *Q*, *R)*. The Figure 5 shows the QCA representation of this gate.

### TR gate

**Figure 6:**
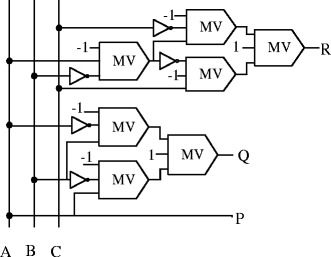
**Proposed QCA block diagram of TR gate.**

The TR gate is a 3-input, 3-output, reversible gate (Thapliyal and Ranganathan 2009b) having inputs to output mapping as $P = A,\ Q=A\oplus B\  and\ R=\left(A\overline{B}\right)\oplus C$, where *A*, *B*, *C* are the inputs and *P*, *Q*, *R* are the outputs, respectively, as shown in Figure 6.

### R gate

**Figure 7:**
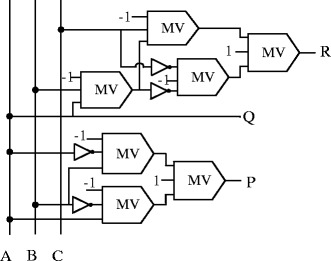
**Proposed QCA block diagram of R gate.**

The R gate is a 3-input, 3-output, reversible gate (Vasudevan et al. 2006). Figure 7 shows the block diagram of this gate in QCA. The input vector is ***I****(A, B, C)* and the output vector is ***O****(P, Q, R).* The outputs are defined as $P=A\oplus B,\ Q=A\  and\ R= AB\oplus \overline{C}$.

### BVF gate

**Figure 8:**
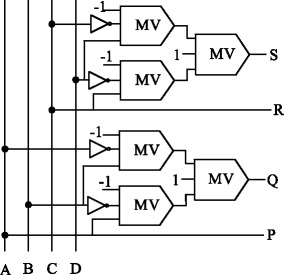
**Proposed QCA block diagram of BVF gate.**

BVF gates also known as 4 × 4 double XOR reversible logic gates (Bhagyalakshmi and Venkatesha 2010). This can be used for duplication of the required inputs to meet the fan-out requirements. The input vector is ***I*** (*A*, *B*, *C*, *D*), the output vector is ***O*** (*P*, *Q*, *R* and *S*) and the output is defined as *P* = *A*, *Q* = *A* ⊕ *B*, *R* = *C and S* = *C* ⊕ *D* shown in Figure 8.

## Simulations and result analysis

**Figure 9:**
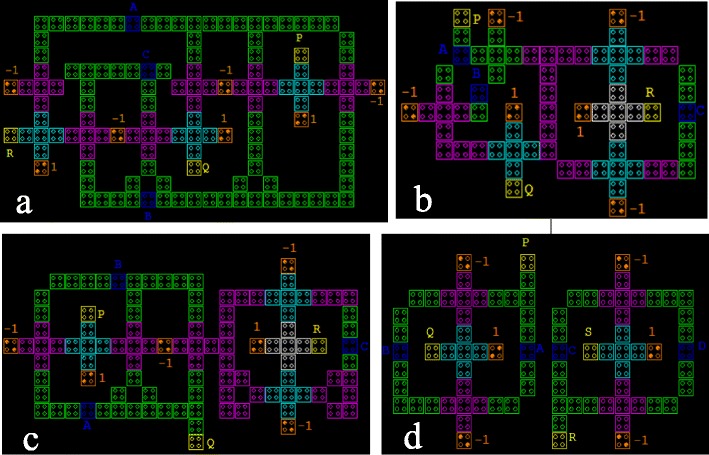
**QCA simulated circuit layout of (a) NFT gate, (b) TR gate, (c) R gate, and (d) BVF gate.**

**Figure 10:**
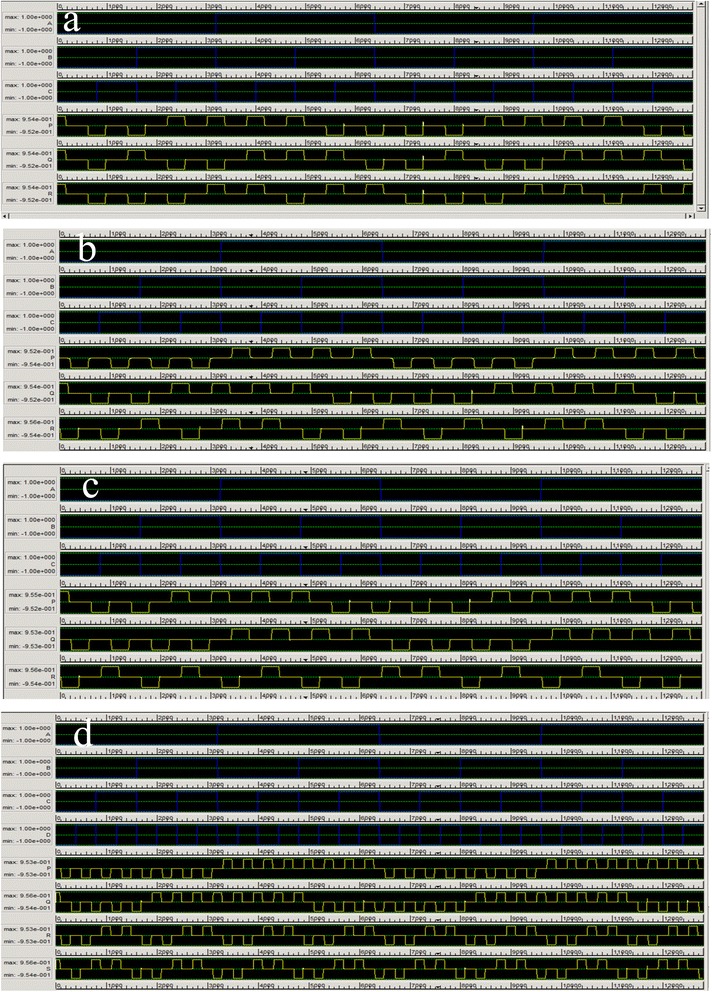
**Input output waveforms of (a) NFT gate, (b) TR gate, (c) R gate, and (d) BVF gate.**

Our proposed circuits have been simulated using the QCADesigner (Walus et al. 2004) a common and powerful simulation tool for QCA circuits. Bistable Approximation has been applied for simulating the proposed circuit with below parameters: cell size = 18 nm, number of samples = 50000, convergence tolerance = 0.0000100, radius of effect = 65.000000 nm, relative permittivity = 12.900000, clock high = 9.800000e^−022^ J, clock low = 3.800000e^−023^J, clock shift = 0, clock amplitude factor = 2.000000, layer separation = 11.500000 and maximum iterations per sample = 100. Most of the above mentioned parameters are default for Bistable Approximation. The circuit layout of NFT, TR, R and BVF gates are shown in Figure 9. Here, the input cells are denoted by A, B, C and D, output cells are P, Q, R and S; and the two polarizations, *P = +1* is denoted by 1 and *P = -1* denoted by -1. Figure 10 shows the input and output waveforms of our proposed gate in QCADesigner.

**Figure 11:**
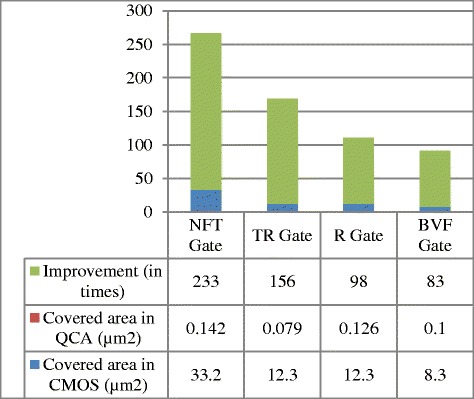
**Comparative figures for covered area (size) of QCA and CMOS with improvement.**

Table 1 shows the different parameters of the proposed gates. From the above table it is clear that QCA technology provides highly integrated designing paradigm over CMOS technology. Covered areas in both CMOS and QCA technologies with improvements are shown in Figure 11. Here, Microwind and Dsch3 has been employed to design and calculate covered area for CMOS design. Moreover, the number of cells and majority voter gates are the total number of cells and majority voter gates required to design a gate.

**Table 1:** **Performance analysis of proposed gates**

**Parameters**	**NFT gate**	**TR gate**	**R gate**	**BVF gate**
Number of cells	128	68	105	82
Number of majority voter gate	9	6	6	6
Time delay (clock cycle)	0.5	0.75	0.75	0.5
Covered area (size) in QCA (μm^2^)	0.142	0.079	0.126	0.10
Covered area (size) in CMOS (μm^2^)	33.02	12.3	12.3	8.3
Improvement (in times)	233	156	98	83

## Conclusion

Quantum-dot cellular automata, one of the promising nanotechnologies that are appropriate for the design of highly scalable logic circuits. A number of QCA-based reversible logic gates, which are significantly smaller size than CMOS have been presented here. In addition, QCA design accomplished by the basic gate and logic circuit in which less area is required to make a device. Thus the new device will consume less power and increase device performance. Since nanotechnology has high demand in the market, this QCA technology can be best suited substitute of CMOS based technology.
